# Superabsorbent polymer balls as foreign bodies in the nasal cavities of children: our clinical experience

**DOI:** 10.1186/s12887-021-02740-x

**Published:** 2021-06-11

**Authors:** Sai-hong Han, Yong-chao Chen, Zhi-xiong Xian, Yi-shu Teng

**Affiliations:** 1 Department of Otorhinolaryngology, Shenzhen Children’s Hospital, 7019 Yitian Road, Futian District, Shenzhen, 518038 Guangdong China; 2 Department of Otorhinolaryngology, Shenzhen Children’s Hospital, China Medical University, Shenzhen, 518038 Guangdong China

**Keywords:** Nasal foreign body, Superabsorbent polymer balls, Nasal injury, Children

## Abstract

**Objective:**

To summarize the clinical diagnosis and treatment of superabsorbent polymer balls as nasal foreign bodies in children.

**Methods:**

We retrospectively analysed the clinical data concerning 12 cases of superabsorbent polymer balls as nasal foreign bodies in children and summarized the corresponding clinical features, methods of diagnoses and treatment, and prognoses.

**Results:**

Twelve children with superabsorbent polymer balls as foreign bodies in their nasal cavities presented with relatively severe symptoms, such as congestion, runny nose, and nasal swelling. When such foreign bodies stay in the nasal cavity for a prolonged period, patients may suffer from general discomfort, such as agitation, poor appetite and high fever. Most of the children had to undergo nasal endoscopy under general anaesthesia to have the foreign bodies completely removed. An intraoperative examination revealed significant mucosal injury within the nasal cavity. With regular follow-up visits and adequate interventions, all the patients recovered.

**Conclusion:**

The longer superabsorbent polymer balls remain in the nasal cavity, the more damaged the nasal mucosa will be. It is challenging to remove such foreign bodies in the outpatient setting. Transnasal endoscopy under general anaesthesia appears to be safer and more effective in such cases. Since the nasal mucosa is injured to varying degrees, postoperative follow-up and treatment are equally important for preventing the occurrence of complications.

## Introduction

Nasal foreign bodies are a common emergency in the paediatric otolaryngology department. Most foreign bodies in paediatric patients are discovered promptly due to symptoms such as nasal congestion and runny noses, and they are largely treatable in the outpatient setting [1]. However, some specific nasal foreign bodies may cause severe damage to the nasal cavity and are associated with significant complications and sequelae [2, 3]. In recent years, toys with superabsorbent polymer balls made of polyacrylate have become increasingly popular. Such foreign bodies have unique physicochemical properties that make the clinical diagnostic and treatment processes especially difficult. From January 2015 to December 2019, our department admitted 12 patients with superabsorbent polymer balls in their nasal cavities. We analysed the clinical diagnostic and treatment data of those patients with the aim of summarizing our experience and providing insights into the future clinical diagnosis and treatment of similar nasal foreign bodies.

## Patients and methods

### General data

Clinical data concerning 12 cases of superabsorbent polymer balls as nasal foreign bodies in patients admitted to the Department of Otolaryngology Head and Neck Surgery, Shenzhen Children’s Hospital, within the 5 years from January 2015 to December 2019 were retrospectively collected. Among the admitted patients, 7 were male and 5 were female. The patients were aged from 1 year and 10 months to 6 years and 9 months (median age: 3 years and 5 months). Eight patients had foreign bodies in the right nasal cavity, whereas 4 had foreign bodies in the left nasal cavity.

### Diagnosis and treatment

Patients suspected of having nasal foreign bodies underwent transnasal endoscopic examination or nasal CT for confirmation when necessary. Once detected, the nasal foreign bodies were removed under local anaesthesia in the outpatient setting, if possible; otherwise, the patients were hospitalized and underwent foreign body removal through transnasal endoscopy under general anaesthesia. After the operation was performed, symptomatic treatment was administered together with regular follow-up visits until complete recovery was achieved.

### Research methods

This paper analyses the clinical symptoms, methods of diagnoses and treatment and postoperative nasal cavity recovery of the involved patients.

## Results

### Clinical data

Twelve patients had nasal foreign bodies that remained in place for 4 h-7 d: 4 patients retained the balls for less than 1 d, 3 retained them for 1–3 d, and 5 retained them for 3–7 d. The clinical manifestations were as follows: the 4 patients who had the foreign bodies lodged in their nasal cavities for less than 1 day had a definite history of nasal foreign bodies, and went to visit the doctor in a timely manner with the symptoms of nasal congestion and a runny nose; the 3 patients who retained the foreign bodies for 1–3 d had severe nasal congestion, snorting, nasal pain, agitation, and refusal to eat; and the 5 patients who retained the foreign bodies for longer than 3 d, had not only common symptoms like nasal congestion, runny nose, agitation and refusal to eat but also nasal swelling and bleeding (2 patients) and a high fever (4 patients).

### Auxiliary examinations

Nasal endoscopic findings were sufficient to confirm the existence of nasal foreign bodies in these 12 patients. Because such foreign bodies may cause severe irritation to the nasal mucosa after absorbing water and expanding in size, the patients had significant swelling of the nasal mucosa. Moreover, the foreign bodies were transparent, which necessitated careful examination to avoid missing the correct diagnosis.

### Treatments

All patients first received local anaesthesia in the outpatient department, and an attempt was made to remove the foreign bodies. If it was impossible to remove the foreign bodies in the outpatient setting, the patients were hospitalized and underwent nasal endoscopy under general anaesthesia. One patient (< 1 d) had the foreign bodies completely removed under local anaesthesia in the outpatient department; 2 patients had the foreign bodies partly removed in the outpatient department under local anaesthesia but experienced nasal congestion, runny nose and a high fever after being sent home and were found to have remaining foreign bodies on nasal endoscopy; thus, the remaining foreign bodies were removed under general anaesthesia. The remaining patients could not be treated in the outpatient department, so they were hospitalized and underwent nasal endoscopy under general anaesthesia. During the operation, the foreign bodies were found to have expanded significantly. As they were highly brittle, they were prone to breaking into pieces when touched. Thus, it was impossible to remove them with hooks or forceps; instead, suctioning was performed multiple times. In patients with foreign bodies left in the nasal cavity for less than 3 d, the nasal mucosa showed local swelling and slight erosion. In those who retained the nasal foreign bodies for longer than 3 d, the nasal mucosa was significantly swollen, with congestion and erosion and a high chance of bleeding. In those patients, more structures were involved, including the inferior nasal concha, nasal septum, concha nasalis media and even the olfactory cleft. In some of the patients, the nasal mucosa had turned ash black, with a false membrane. However, no perforation of the nasal septum was found in any patient. During the operation, after all the foreign bodies had been removed, the nasal secreta, surface false membranes and necrotic tissues were carefully cleaned, and the nasal cavity was repeatedly washed with normal saline.

### Postoperative follow-up

After the operation, patients were treated with anti-inflammatories and antibiotics based on their nasal cavity injury. Saline was used to clean the nasal cavity regularly. Those with slightly injured nasal mucosa recovered within 1 month after the operation, with granulation within 2 months. The nasal mucosa was covered with a large quantity of dry yellow crust, which necessitated repeated cleaning and treatment. The patients mostly recovered within 3 months. Only one patient had an adhesion within the nasal cavity within 6 months after the operation, and the nasal adhesion was lysed under general anaesthesia. The nasal cavity in this patient recovered within 9 months.

### Typical case

**Fig. 1:**
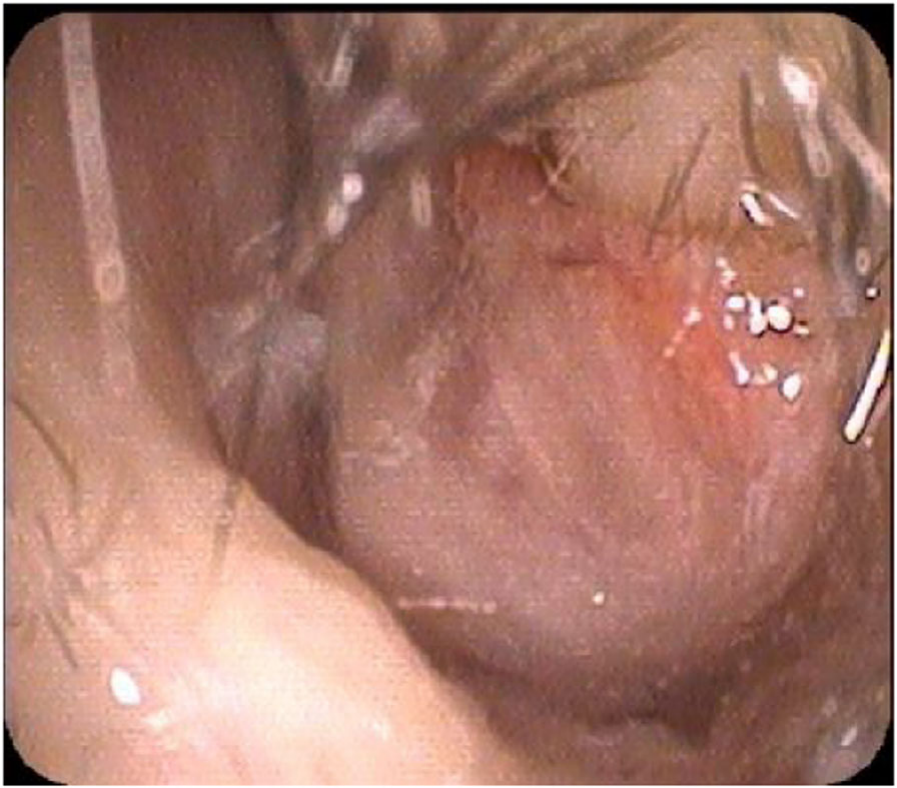
Nasal endoscopic examination: swollen inferior nasal concha in the left, greyish white foreign bodies in the common meatus

**Fig. 2:**
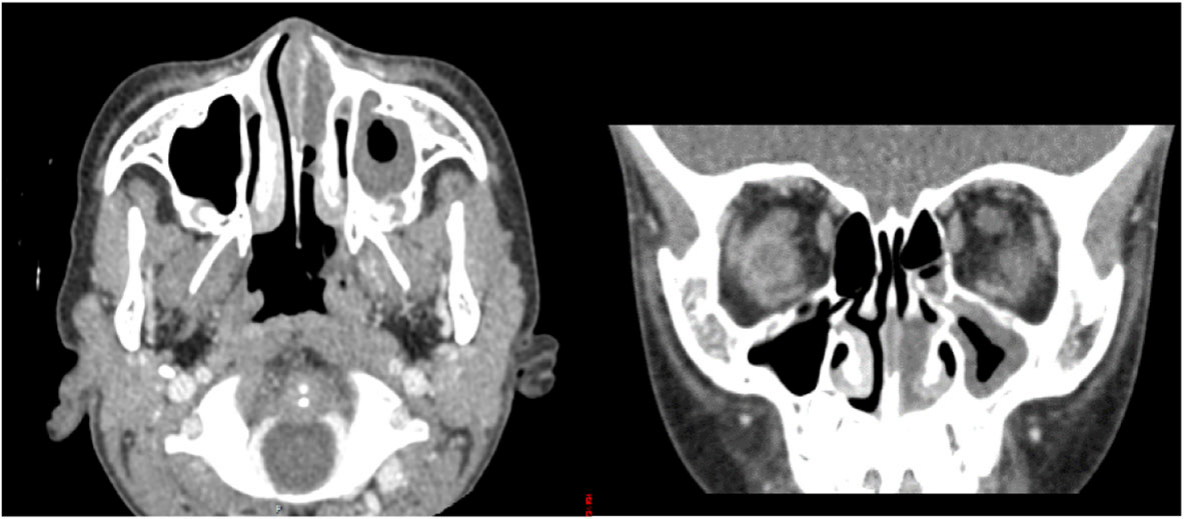
Foreign bodies measuring 27 mm × 13 mm with uniform density and inflammation of the left maxillary sinus and ethmoidal sinus

**Fig. 3:**
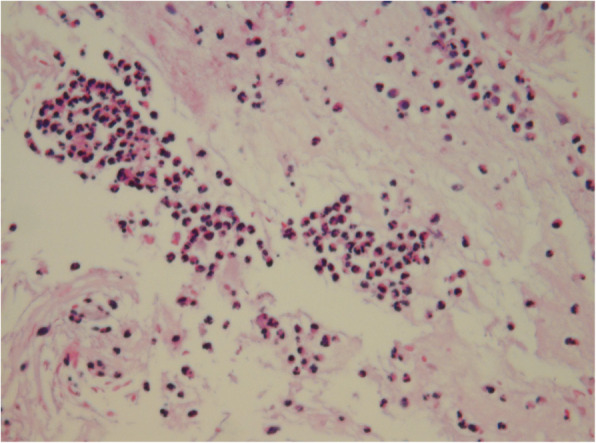
Pathological examination suggested cellulose-like effusion combined with inflammatory necrotic changes and substantial neutrophil infiltration (HE× 200)

A 4-year-and-6-month-old male patient, was admitted due to swelling of the left nasal cavity for 3 days. Nasal endoscopy revealed a severely swollen left nasal cavity with an occupying greyish white substance (Fig. 1). When asked about the child’s medial history, the parents could not provide any information concerning the child’s contact with foreign bodies. Due to his significantly swollen nasal cavity, the patient cried, screamed and refused to cooperate with the removal process. Thus, he was recommended to be hospitalized for further diagnosis and treatment under general anaesthesia. However, the recommendation was refused by his parents. Two days later, the child was suffering from worse pain in his nasal cavity, accompanied by nasal bleeding and a high fever. He returned to the hospital. After being hospitalized, the child underwent a nasal CT examination, which suggested the same foreign object (26.6 mm × 13 mm) in the common meatus of the left nasal cavity combined with inflammation of the left maxillary sinus and ethmoidal sinus (Fig. 2). A biopsy of the space-occupying lesion was performed, which indicated cellulose-like effusion combined with inflammatory necrotic changes and substantial neutrophil infiltration (Fig. 3). Nasal endoscopic examination under general anaesthesia supported the identification of the foreign bodies as greyish white superabsorbent polymer balls that had expanded significantly, impinging on the nasal mucosa. The nasal mucosa appeared very swollen and eroded on the surface, with a white false membrane. Postoperative inquiry into the patient’s history revealed that the patient had come into contact with toys containing such superabsorbent polymer balls in a shopping mall 1 week prior. Regular follow-up visits were arranged after the operation. Three months later, the patient’s nasal mucosa had recovered to normal, and his naso-sinusitis was cured.

## Discussion

Nasal foreign bodies in children are commonly seen in clinical practice. Children cannot report their medical history and their parents are often unaware of the precipitating incident; therefore, misdiagnosis and missed diagnosis are common [4]. When foreign bodies remain in the nasal cavity for a prolonged period, they can cause chronic diseases such as rhinitis and upper airway cough syndrome (UACS) [5, 6]. Nasal foreign bodies becoming tracheal foreign bodies is very rare, but they may be accidently sucked into the trachea, posing a severe threat to the life of the patient [7, 8]. Once the presence of nasal foreign bodies has been confirmed, they should be removed as soon as possible.

**Fig. 4:**
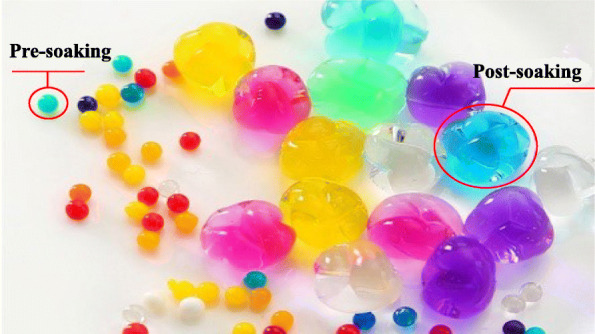
After being soaked in water for 30 min, the ball (diameter: 2 mm) becomes enlarged, with a diameter of 10 mm

Children are increasingly exposed to a variety of objects, such as stationery, toys and food, leading to a higher incidence of nasal foreign bodies [9]. As the physicochemical properties of these foreign bodies tend to be increasingly diversified and complicated [5], it has become even more challenging to remove them from the nasal cavity. In addition, multiple complications and sequalae may develop. Superabsorbent polymer balls have become common foreign bodies in recent years. They usually appear in the form of soft marbles that are the projectiles associated with toy guns that are accessible to children. They strongly resembling candies, with their smooth and either transparent or colourful appearance, and they have become very popular among children (Fig. 4). Superabsorbent polymer balls are now frequently reported as causing digestive tract obstruction after being accidently eaten by children [10, 11], and they have also been reported as a cause of injury to the ear or even death in severe cases [12].

Superabsorbent polymer balls are mainly made of polyacrylate-polyacrylamide copolymer and are able to absorb water. When exposed to water, they can expand to several times their original size. Therefore, they usually impinge on the nasal cavity, causing ischaemia and necrosis of the nasal mucosa. Furthermore, acrylamide is neurotoxic in nature. Although it loses its toxicity after being polymerized, it could still cause chemical corrosion of the nasal mucosa once introduced and may even enter the digestive tract and respiratory tract, where it is absorbed, entering the systemic circulation and causing toxic reactions.

The patients in this study retained the foreign bodies for less than 1 week; however, they presented with relatively severe clinical manifestations, such as nasal congestion, snorting and runny nose, than those with other kinds of nasal foreign bodies. Some of the patients in this study suffered from severe symptoms, such as swelling of the nasal cavity and nasal bleeding. When the foreign bodies remained in the nasal cavity for a prolonged period, the patients developed agitation, refusal to eat and a high fever. Intraoperative examination confirmed severe injury to the nasal mucosa combined with congestion, swelling and the development of a false membrane resulting from impingement, ischaemia and necrosis.

Superabsorbent polymer balls are prone to breaking into gel particles when touched. They are not only brittle but also too slimy to be gripped. Thus, they have to be suctioned out of the nasal cavity one at a time. Due to the presence of the foreign bodies, the patients had severe inflammation in the nasal cavity, resulting in swelling of the mucosa and the consequent obstruction of the visual field. Moreover, the children often do not cooperate during the removal of the foreign bodies. When foreign bodies could not be removed under local anaesthesia, general anaesthesia was needed to enable clinicians to remove the foreign bodies during nasal endoscopy. In the patients in this study, the longer the foreign bodies were retained, the more severe the clinical manifestations. Severe damage of the mucosa was confirmed on endoscopy. This justifies the decision to remove foreign bodies such as superabsorbent polymer balls from the nasal cavity by performing nasal endoscopy under general anaesthesia after the failure of attempts to remove them under local anaesthesia.

Patients with such nasal foreign bodies usually experience nasal mucosal injury to varying degrees, and it takes time for the mucosa to recover after the operation. Patients should clean their nasal cavity with normal saline on a regular basis. General and local anti-inflammatory and anti-infection therapies should be administered. Within 1–2 months after the operation, abnormalities such as dry crust and granulation may be expected, which should be detected and removed promptly.

## Conclusion

Superabsorbent polymer balls are unique nasal foreign bodies that can cause severe harm to the nasal mucosa if left for a prolonged period. Patients which such foreign objects may display more significant symptoms than those with common nasal foreign bodies, including nasal congestion, runny nose and nasal swelling. In severe cases, the patients may become agitated, refuse to eat and develop a high fever. When patients with such symptoms appear in the clinic, there should be a strong suspicion of the presence of these unique nasal foreign bodies. The foreign bodies should be removed safely and effectively to prevent complications and further nasal injury. Regular postoperative follow-up is needed to ensure prompt interventions to address sequalae such as nasal cavity adhesions.

## Data Availability

The datasets used and/or analysed during the current study are available from the corresponding author (YS.T, tys118@163.com) on reasonable request.
